# Quercetin Alleviates High-Fat Diet-Induced Oxidized Low-Density Lipoprotein Accumulation in the Liver: Implication for Autophagy Regulation

**DOI:** 10.1155/2015/607531

**Published:** 2015-12-01

**Authors:** Liang Liu, Chao Gao, Ping Yao, Zhiyong Gong

**Affiliations:** ^1^College of Food Science and Engineering, Wuhan Polytechnic University, Wuhan 430023, China; ^2^Hubei Collaborative Innovation Center for Processing of Agricultural Products, Wuhan Polytechnic University, Wuhan 430023, China; ^3^National Institute for Nutrition and Health, Chinese Center for Disease Control and Prevention, Beijing 100050, China; ^4^Department of Nutrition and Food Hygiene, Hubei Key Laboratory of Food Nutrition and Safety, School of Public Health, Tongji Medical College, Huazhong University of Science and Technology, Wuhan 430030, China

## Abstract

A growing body of evidence has indicated that high-fat diet-induced nonalcoholic fatty liver disease is usually accompanied by oxidized low-density lipoprotein (ox-LDL) deposited in the liver. The current study aimed to investigate the effect of quercetin on high-fat diet-induced ox-LDL accumulation in the liver and to explore the potential underlying mechanisms. The results demonstrate that quercetin supplementation for 24 weeks significantly alleviated high-fat diet-induced liver damage and reduced hepatic cholesterol and ox-LDL level. Quercetin notably inhibited both mRNA and protein expression of CD36 (reduced by 53% and 71%, resp.) and MSR1 (reduced by 25% and 45%, resp.), which were upregulated by high-fat diet. The expression of LC3II was upregulated by 2.4 times whereas that of p62 and mTOR was downregulated by 57% and 63% by quercetin treatment. Therefore, the significantly improved autophagy lysosomal degradation capacity for ox-LDL may be implicated in the hepatoprotective effect of quercetin; scavenger receptors mediated ox-LDL uptake might also be involved.

## 1. Introduction 

Nonalcoholic fatty liver disease (NAFLD), one of the most common causes of chronic liver disease, ranging from steatosis (simple fatty liver) to nonalcoholic steatohepatitis (NASH) and to advanced fibrosis and cirrhosis, is determined by multiple factors or “hits” which may act in sequence or in parallel [1]. It has been proven that lipid accumulation, inflammatory process, and insulin resistance (IR) appear to be crucial in the onset of NAFLD [2]; nevertheless, emerging findings pointed out an important role for modified lipoprotein, especially oxidized low-density lipoprotein (ox-LDL) [3]. While the key role of ox-LDL in the etiology of atherosclerosis and coronary artery disease has been extensively researched owning to its implication in the formation of foam cells and macrophage apoptosis [4], the potential pathological significance of ox-LDL in NAFLD has not been clarified thus far [5].

Recently, Bieghs and his colleagues reported the presence of bloated Kupffer cells (KCs) in hyperlipidemic mice fed with a western diet [6, 7] and lysosomal trapping of ox-LDL in KCs with concomitant hepatic inflammation in Ldlr^−/−^ mice exposed to ox-LDL via tail vein injection [8]; nonalcoholic steatohepatitis in mice reduced with specific immunization strategies against ox-LDL [3]. Therefore, it has been considered that hepatic inflammatory response attributed to trapped ox-LDL in lysosomes of KCs is mediated by CD36 and SR-A (also known as macrophage scavenger receptor 1, MSR1) [6], although deletion of CD36, MSR1, or both did not completely abrogate foam cell formation in vitro or in vivo.

In addition to the evolved homeostatic mechanisms for regulating transport and storage of ox-LDL, mobilization of ox-LDL is also involved in cellular defenses against ox-LDL toxicity. However, unlike acetylated LDL (acLDL) that normally undergoes lysosomal hydrolysis resulting in its accumulation in the cytoplasmic storage of cholesteryl esters, ox-LDL is resistant to hydrolysis by lysosomal proteinases and alters the activity of the ubiquitin-proteasome pathway leading to its accumulation within macrophages [9]. Therefore, it has been proposed that autophagy provides a possible alternate pathway for clearing aggregated ubiquitinated proteins when the proteasome is impaired [10]. In line with this view, Zhang et al. found that the autophagy lysosome pathway was involved in the degradation of ox-LDL in human vascular endothelial cells [11]. However, it is still questionable whether autophagy lysosome pathway is implicated in ox-LDL degradation in the liver and is involved in the progression of NAFLD.

Quercetin is the most widely distributed flavonoids occurring ubiquitously in plant-derived foods and accounts for 60–75% of the total flavonol plus flavone intake [12–15]. Quercetin has attracted increasing attention for its broad spectrum of beneficial health effects against various diseases. Growing experimental data have demonstrated that quercetin could be explored as a potential candidate for the prevention of NAFLD [16] since it has an important role in maintaining homeostatic balance of redox status [17–19], ameliorating inflammation and fibrosis [16, 20], regulating the expression of lipid metabolism genes [21, 22], and improving liver integrity [23]. To the best of our knowledge, however, little attention has been focused on the effect of quercetin on the mobilization of ox-LDL mediated by autophagy lysosomal pathway in a long-term high-fat diet- (HFD-) induced mouse model. The ability of quercetin to induce autophagy has been extensively studied in different cancer models in vivo and in vitro [24–26]. Herein, we investigated the potential protective role of quercetin on HFD-induced liver damage by focusing on ox-LDL and autophagy regulation.

## 2. Materials and Methods

### 2.1. Chemicals and Materials

Quercetin (≥98%, HPLC) and sodium dodecyl sulfate (SDS) were provided by Sigma-Aldrich (St. Louis, Missouri, USA). Anti-CD36 rabbit polyclonal antibody (ab78054) and anti-LC3 A/B rabbit polyclonal antibody (ab58610) were obtained from Abcam (Massachusetts, USA). Anti-GAPDH rabbit polyclonal antibodies were purchased from Boster Biological Technology, Ltd. (Wuhan, China). Anti-MSR1 rabbit monoclonal antibody (P21757) was obtained from Epitomics (Burlingame, CA, USA). Anti-ox-LDL rabbit polyclonal antibodies (bs-8574R) were provided by Biosynthesis Biotechnology CO., Ltd. (Beijing, China). Anti-mTOR (7C10) rabbit monoclonal antibody (#2983), anti-SQSTM1/p62 rabbit polyclonal antibodies (#5114), horseradish peroxidase- (HRP-) conjugated anti-rabbit IgG (secondary antibody, #7074), and HRP-conjugated anti-mouse IgG (secondary antibody, #7076) were obtained from Cell Signal (Beverly, MA, USA). Western blotting detecting reagents (ECL) and reblot buffer were provided by Chemicon (Temecula, CA, USA). Detection kits for total cholesterol (TC) and triglyceride (TG), were purchased from Biosino Biotechnology Co., Ltd. (Beijing, China). Other chemicals and organic solvents were of analytical grade and purchased from local reagent retailer.

### 2.2. Animal Treatment

Animals were cared for according to the Guide for the Care and Use of Laboratory Animals (Institute of Laboratory Animal Resources, Commission on Life Sciences, National Research Council, 1996). Experiments described in this study were approved by the Tongji Medical College Council on Animal Care Committee. Forty-five healthy male ApoE-knockout (C57BL/6J background) mice weighing 16–18 g, purchased from Vital River Laboratory Animal Technology Co., Ltd. (Beijing, China), were randomly divided into three groups of fifteen animals each. The three groups of animals were fed with normal chow diet (ND group), HFD (containing 21% fat + 1.25% cholesterol, HFD group) [27], and HFD plus quercetin (100 mg/kg·bw, HFD + QR group), respectively. Meanwhile, fresh water was provided* ad libitum* and body weight was monitored once a week. Animals were kept on a regular 12 : 12 light dark cycle at a controlled temperature (24 ± 2°C) and relative humidity (65–75%). The mice were sacrificed after overnight fasting. Serum was collected from blood by centrifugation at 3500 g for 10 min at 4°C (Eppendorf 5810R, Hamburg, Germany). Fresh liver specimens were quickly weighted, frozen by liquid nitrogen, and stored at −80°C for various assays.

### 2.3. Histological and Immunohistochemistry Analysis

Liver tissues removed aseptically from the animals were fixed in 4% paraformaldehyde/phosphate-buffered saline, and then the specimens were embedded in paraffin and cut into thin slices (5 *μ*m). The slices were stained with hematoxylin and eosin (H&E) or incubated with anti-ox-LDL antibody (1 : 100 dilution) overnight at 4°C. Hepatic histopathological changes were detected by light microscope. Immunostaining was visualized with 3,3′-diaminobenzidine following the reaction with corresponding secondary antibody at 1 : 200 dilution for 1 h.

### 2.4. Determination of Hepatic TC and TG

Hepatic TC and TG content were determined according to a previously described method [28]. Briefly, liver homogenates (10%) were prepared with isopropanol and stewed for 48 h at 4°C. Following centrifugation at 3000 rpm for 15 min at 4°C, the supernatants were collected to measure hepatic TC and TG content by using commercial kits according to the manufacturer's recommendations and the results were normalized to total protein detected by the method of Lowry [29].

### 2.5. Real-Time Quantitative Polymerase Chain Reaction (PCR) Analysis

Expression of mRNA was determined using real-time reverse transcription polymerase chain reaction (RT-PCR). Total RNA was extracted from liver tissue using the Trizol reagent (Invitrogen, Carlsbad, CA, USA) according to the manufacturer's instructions. mRNA expressions of the target genes were quantified by quantitative reverse transcriptase- (qRT-) PCR using the SYBR green-based kit (TaKaRa BIO Inc., Dalian) with specific primers using an RT-PCR machine (7900HT; Applied Biosystems, Forster, CA, USA). The forward and reverse primers for CD36 were CGG GCC ACG TAG AAA ACA CT and CAG CCA GGA CTG CAC CAA TA, respectively. The primers of MSR1 were GAC TTC GTC ATC CTG CTC AAT and GCT GTC GTT CTT CTC ATC CTC. The primers of *β*-actin were TTC GTT GCC GGT CCA CAC CC and GCT TTG CAC ATG CCG GAG CC. The mRNA level of *β*-actin was quantified as an endogenous control, and results were calculated by a comparative 2^−ΔΔCt^ method.

### 2.6. Western Blot Analysis

Mouse liver tissues were minced and homogenized in radio-immunoprecipitation assay (RIPA) lysis buffer (1% Triton X-100, 1% deoxycholate, and 0.1% sodium dodecyl sulfate (SDS)) containing 1% (v/v) protease inhibitor phenylmethanesulfonyl fluoride (PMSF). The homogenates were centrifuged at 10,000 g for 15 min at 4°C and protein concentrations were determined. Equal amounts of protein extracts were mixed (3 : 1) with loading buffer for electrophoresis in 10%–12% acrylamide SDS gels and subsequently electroblotted to polyvinylidene fluoride membrane (PVDF) (Millipore, MA, USA) by electrophoresis (Bio-Rad, USA). Target proteins were probed with the specific primary antibodies against the target protein and then incubated with the species-specific second antibodies conjugated to HRP. The chemiluminescence intensity of membrane was subsequently detected by ECL Plus kit with Western Blotting Detection System (Amersham Biosciences, Little Chalford, UK), and optical densities of bands were quantified by Gel Pro 3.0 software (Biometra, Goettingen, Germany). Data were corrected to eliminate background noise and standardized to GAPDH as optical density (OD/mm^2^).

### 2.7. Statistical Analysis

All data were entered into Excel and analyzed by SPSS 12.0 software package using one-way analysis of variance test. The data were expressed as means ± SEM and were considered significantly different at *P* < 0.05 and *P* < 0.01.

## 3. Results

After 24 weeks of feeding, body weight and liver ratio to body weight were recorded and the results are shown in Table 1. HFD markedly affected final body weight and ratio of liver to body weight in comparison with ND. However, quercetin supplementation had no influence on body weight and liver weight in comparison with HFD, suggesting that quercetin consumption had no effect on body weight gain and ratio of liver to body weight.

### 3.1. Effects of Quercetin on HFD-Induced Pathological Changes and Hepatic TC and TG Levels

**Figure 1:**
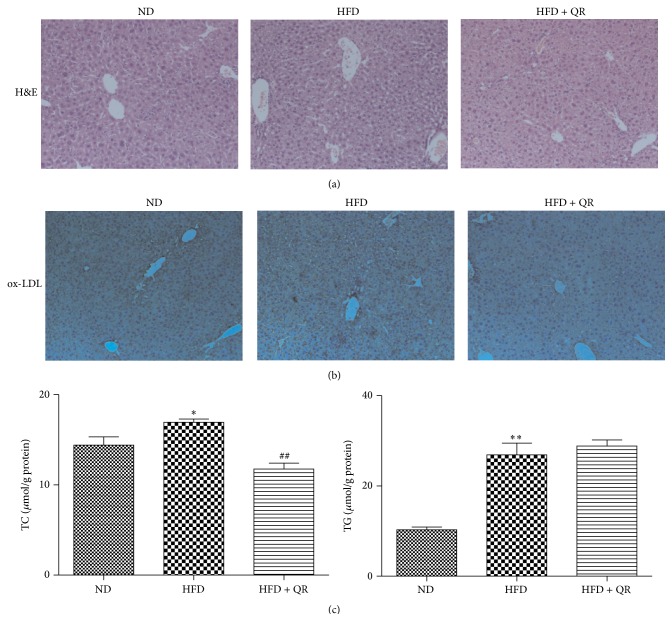
Effects of quercetin on hepatic histological changes and hepatic lipid content in mice fed with high-fat diet. Fixed liver tissue sections (three independent experiments) of mice (a) stained with H&E and (b) stained with anti-ox-LDL were observed under light microscope (magnification 40x); (c) hepatic lipids levels. ^*∗*^ *P* < 0.05 versus ND group, ^*∗∗*^ *P* < 0.01 versus ND group, and ^##^ *P* < 0.01 versus HFD group (*n* = 10 for hepatic lipids levels).

H&E stained and anti-ox-LDL immunostained liver tissue sections are shown in Figure 1. Mice fed with an HFD for 24 weeks displayed marked symptoms of hepatic fatty infiltration compared to ND-fed mice, but long-term dietary quercetin supplementation significantly alleviated these symptoms. Consistent with histopathological examination, hepatic TC and TG levels also increased in HFD-fed mice. Daily quercetin supplementation reduced hepatic TC, but not TG, accumulation in HFD-fed mice compared to mice receiving HFD alone.

Hepatic ox-LDL deposition was identified immunohistochemically with anti-ox-LDL antibody. As illustrated, quercetin reduced hepatic ox-LDL deposition, indicating the effects of quercetin on metabolism of ox-LDL. However, no apparent difference was observed in ox-LDL-positive staining between ND and HFD groups, although the amount of lipid vacuoles in HFD group was more than that in the ND group; similar results were observed with H&E staining, which indicated that ox-LDL might contribute to hepatic fatty infiltration. Distinct pathologic characteristics and inconspicuous differences in ox-LDL level suggested that hepatic ox-LDL accumulation and histopathological changes induced by HFD are implicated in several processes, such as uptake and/or degradation of ox-LDL.

### 3.2. Effect of Quercetin on Expression of Hepatic Scavenger Receptors

**Figure 2:**
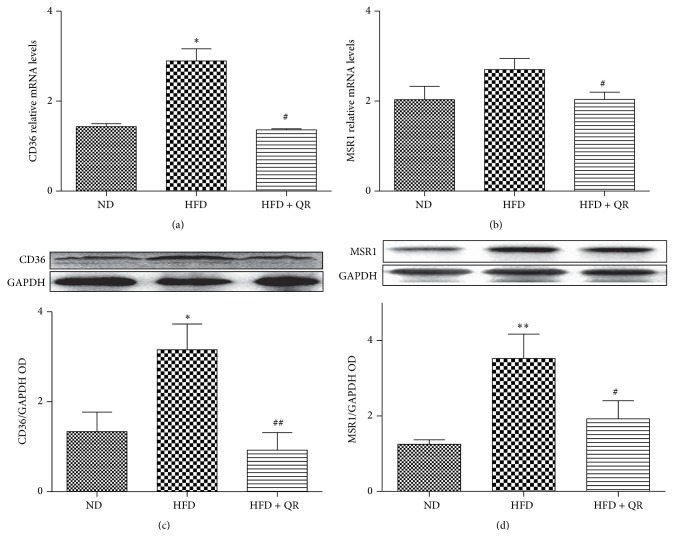
Effects of quercetin on the expression of CD36 and MSR1 in the livers of mice fed with high-fat diet. Hepatic mRNA expression of CD36 (a) and MSR1 (b) was measured by RT-PCR. Protein levels of CD36 (c) and MSR1 (d) were determined with western blot analysis. Bar diagrams show densitometric data (c and d). Results expressed as mean ± SEM. ^*∗*^ *P* < 0.05 versus ND group, ^*∗∗*^ *P* < 0.01 versus ND group, ^#^ *P* < 0.05 versus HFD group, and ^##^ *P* < 0.01 versus HFD group (*n* = 5 for RT-PCR in each group; *n* = 3 for others).

To determine the protective role of quercetin on exaggerated liver damage induced by HFD, we measured the mRNA and protein expression of CD36 and MSR1, two main scavenger receptors involved in ox-LDL uptake, by RT-PCR and western blot analysis. In contrast to HFD-fed mice, mice receiving daily quercetin supplementation had significantly decreased mRNA and protein expression of both CD36 and MSR1. These results indicated that quercetin might be a promising inhibitor of scavenger receptors under an HFD condition for relieving liver damage induced by excessive ox-LDL accumulation. Nevertheless, the lower expression of CD36 and MSR1 and ox-LDL deposition in ND-fed mice was comparable to that observed in HFD-fed mice. Thus, we presumed that the liver damage induced by HFD not only is involved in ox-LDL uptake but also may partially implicated in ox-LDL degradation in liver tissue (Figure 2).

### 3.3. Quercetin Altered HFD-Induced Protein Expression of LC3II, p62, and mTOR

**Figure 3:**
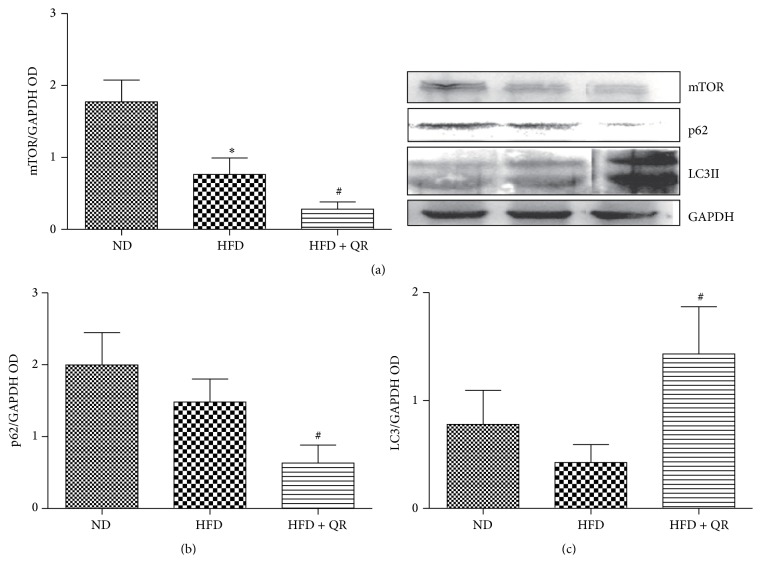
Quercetin supplementation increased the protein expression of LC3II and further downregulated the protein expression of mTOR and p62 induced by high-fat diet. Bar diagrams show densitometric data (a, b, and c). Results expressed as mean ± SEM. ^*∗*^ *P* < 0.05 versus ND group; ^#^ *P* < 0.05 versus HFD group (*n* = 4 for western blot analysis in each group).

The amount of ox-LDL accumulation observed in HFD-fed mice was comparable to that in ND-fed mice, and to investigate this further, we examined autophagic degradation of lipids, known as lipophagy, which served as a possible alternate pathway for clearing lipoprotein. Thus, the protein expression of autophagy marker protein, microtubule-associated protein 1, light chain 3 beta (LC3II), a selective substrate of the autophagy autophagosome adaptor p62 (also known as SQSTM1), and negative regulator of autophagy-mammalian target of rapamycin (mTOR) were analyzed by western blot. The normalized intensity ratio of LC3II, p62, and mTOR to *β*-actin is summarized in Figure 3. LC3II expression decreased in response to chronic HFD consumption relative to ND group. However, the decreased LC3II expression was effectively reversed by quercetin treatment. These findings implied that autophagy dysfunction was possibly due to long-term HFD-induced excessive ox-LDL accumulation and quercetin improved autophagic flux by increasing the formation of autophagosomes. Similarly, the expression of p62 was low in quercetin treatment group, suggesting that more p62 protein was degraded most likely owing to the increase in autophagic flux induced by quercetin. Meanwhile, expression of mTOR, autophagy negative regulatory protein, showed a similar trend as that of p62. These results supported the view that quercetin played an important role in autophagy regulation under atherogenic HFD condition, presumably by inhibiting the mTOR pathway.

## 4. Discussion 

Apolipoprotein E is required for normal catabolism and clearance of lipoprotein constituents and acts as a ligand for cell-surface LDL receptors. Therefore, apoE-knockout mice (apoE^(−/−)^) experience a severe, progressive form of hypercholesterolemia, making them the most commonly employed model for studying the adverse influence of cholesterol on various body [30]. NAFLD, affecting up to a third of the population in many developed countries [31], has recently attracted considerable attention worldwide. An HFD is widely used to induce hepatic steatosis and NASH in experimental animals. Several studies, including our own, have shown that long-term intake of HFD, which can induce obesity and insulin resistance, induces NASH and liver tumorigenesis in C57BL/6J mice [32].

Increasing amounts of data have shown that NAFLD frequently coexists with metabolic syndrome such as obesity, diabetes, atherosclerosis, and dyslipidemia [33–36]. Therefore, apoE^(−/−)^ mice fed with HFD, the classical animal model for atherosclerosis research, are often employed to explore the mechanism of NAFLD [37–40]. In the present study, apoE^−/−^ mice were fed with HFD containing 1.25% cholesterol and 21% fat with or without quercetin (100 mg/kg bw) for 24 weeks. Our study showed that quercetin supplementation had no effect on final body weight and liver ratio to weight, which was not in agreement with the existing reports [22] possibly owing to the longer feeding period and higher proportion of cholesterol employed in our study. While quercetin reduced the elevated serum lipid (data not shown) and hepatic TC level, it had no effect on the increase in hepatic TG level.

Several mechanisms of ox-LDL contributing to the atherosclerotic plaque formation and progression have been well documented. Ox-LDL acts by binding to several SRs, including SR-A, SR-BI, CD36, and lectin-like oxidized low-density lipoprotein receptor-1 (LOX-1) and transforms macrophages to foam cells, which are a hallmark of atherosclerosis. Recently, increasing evidence is available for hepatic inflammation due to ox-LDL deposition [6, 8]. Mice injected with ox-LDL via tail vein displayed swollen KCs and hepatic inflammation due to ox-LDL accumulation within lysosomes [8]. Hyperlipidemic mice fed with a western diet show an early onset of hepatic inflammation associated with bloated Kupffer cells (KCs) which resemble the foam cells of atherosclerotic lesions [41].

The role of macrophage SRs in atherogenesis has been extensively investigated since SR-A and CD36 degrade 75–90% of acetylated or oxidized LDL [42]. Uptake and internalization of modified LDL by SRs are believed to constitute one of the major pathways of foam cell formation in vivo. Quercetin protects macrophages from ox-LDL-induced lipid accumulation by inhibiting the endoplasmic reticulum stress-C/EBP homologous protein pathway [43], reduces HFD-induced fat accumulation in the liver by regulating lipid metabolism genes, including CD36 [44], and ameliorates inflammation and fibrosis in mice with nonalcoholic steatohepatitis [20]. It has been illustrated that quercetin reduces foam cell formation by downregulating surface expression of the ox-LDL receptor CD36 in ox-LDL-treated mice [45]. The current findings showed that HFD increased and quercetin decreased both the mRNA and protein expression of CD36 and MSR1, indicating the possible protective role of quercetin in HFD-induced liver damage via inhibition of scavenger receptor expression. However, ox-LDL deposition in the liver tissue between ND and HFD groups did not display statistically significant difference, although the CD36 and MSR1 expression showed statistical difference (*P* < 0.05). Scavenger receptors CD36 and MSR1 are responsible for the majority of modified LDL uptake into macrophages; these receptors are unlike the native LDL receptor (LDL-R) in that they are not feedback-controlled. These observations suggested that scavenger receptors modulate inflammation without altering ox-LDL accumulation in the liver. Quercetin not only reduced ox-LDL accumulation but also alleviated inflammatory response induced by HFD. These findings suggested that the degradation pathway for intracellular ox-LDL also contributes to ox-LDL deposition in liver tissue, and quercetin reduced ox-LDL deposition indicating its potential function in ox-LDL degradation.

Autophagy or cellular self-digestion, the basic catabolic mechanism that involves degradation of unnecessary or dysfunctional cellular components through the actions of lysosomes, has been reported to be implicated in a broad spectrum of mammalian diseases. Recently, a growing body of research has shown the correlation between regulation of autophagy and liver complications associated with obesity, NAFLD [46]. However, the role of autophagy in the pathophysiology of NAFLD has been controversial [47]. Under physiological conditions, autophagy is maintained at very low levels and is involved in the degradation of long-lived proteins. The ubiquitin-proteasome pathway, another catabolic process responsible for the degradation of short-lived proteins, has no role in ox-LDL degradation owing to the resistance of ox-LDL to hydrolysis by lysosomal proteinases, indicating that an alternative pathway may be involved in ox-LDL degradation. Zhang et al. found that ox-LDL could activate the autophagic lysosome pathway in human vascular endothelial cells through the LC3/beclin1 pathway, leading to the degradation of ox-LDL [11]. However, the underlying intracellular pathway that contributes to hepatic inflammation has not been established, although a novel animal model [48] showed the direct involvement of ox-LDL in the development of NASH. Therefore, we proposed that the behavior and fate of intracellular ox-LDL are responsible for the pathophysiological symptoms associated with long-term HFD treatment.

Quercetin, a versatile bioactive flavonoid, has been extensively studied in renal ischemia/reperfusion injury [49], leukemia [50], and many cancers [51] for its beneficial effects in autophagy regulation. However, the protective role of quercetin in NAFLD by influencing autophagy regulation has not been investigated directly. While quercetin is involved in the metabolism of unoxidized lipids, there exists no direct evidence to support the role of autophagy in the removal of ox-LDL [11]. Thus far, LC3 has been used as one of the reliable autophagosome markers for monitoring autophagy, and the amount of LC3-II correlates with the number of autophagosomes. As shown in this study, the ox-LDL deposition and severe liver damage induced by HFD were comparable to those induced by ND treatment; however, quercetin treatment not only reduced ox-LDL accumulation but also alleviated liver damage. Our findings showed that long-term HFD intake induced excessive ox-LDL deposition, which possibly further led to impairment of damage. These data indicate that quercetin can protect the liver from HFD-induced ox-LDL deposition partly by improving the autophagy lysosomal signaling pathway. The exact mechanisms of ox-LDL degradation by lysosomal autophagy pathway in HFD-induced NAFLD should be further verified in vitro.

## 5. Conclusion 

In conclusion, quercetin decreased excessive deposition of hepatic ox-LDL and alleviated long-term HFD-induced liver damage. Apart from inhibiting the expression of the scavenger receptors, including MSR1 and CD36 that are mainly responsible for hepatic ox-LDL uptake under HFD conditions, quercetin improved autophagic capacity possibly implicated in ox-LDL degradation mediated by autophagy lysosomal pathway; this might partially contribute to its protective effects. Our findings highlight the role of quercetin or other naturally occurring phytochemicals with autophagy modulation effect in prophylaxis of ox-LDL/HFD-induced liver damage/NAFLD.

## Figures and Tables

**Table 1:** Effects of quercetin on body weight and liver ratio to body weight in mice chronically fed with high-fat diet for 24 weeks.

	ND	HFD	HFD + QR
Initial weight (g)	17.3 ± 1.2	16.7 ± 1.1	16.6 ± 1.3
Final weight (g)	26.8 ± 2.1	29.6 ± 2.1^*∗*^	30.3 ± 1.9^*∗*^
Liver to body weight ratio (%)	3.9 ± 0.4	4.7 ± 0.3^*∗*^	4.8 ± 0.5^*∗*^

ND: ApoE^(−/−)^ mice fed with normal diet; HFD: ApoE^(−/−)^ mice fed with high-fat diet; and HFD + QR: ApoE^(−/−)^ mice fed with high-fat diet and quercetin (100 mg/kg·bw). ^*∗*^ *P* < 0.05 versus ND group (*n* = 12 in each group).
